# Modern contraceptive method utilization and determinant factors among women in Ethiopia: Multinomial logistic regression mini- EDHS-2019 analysis

**DOI:** 10.1186/s40834-023-00235-x

**Published:** 2023-07-25

**Authors:** Berhan Tsegaye Negash, Aklile Tsega Chekol, Mastewal Aschale Wale

**Affiliations:** 1 Department of midwifery, College of medicine and Health sciences, Hawassa University, Hawassa city, Ethiopia; 2 Department of Nursing, College of Medicine and Health sciences, Hawassa University, Hawassa city, Ethiopia

**Keywords:** Modern, Contraceptive, Traditional, Women of Reproductive age group, Ethiopia

## Abstract

**Background:**

Globally, approximately 290,000 women between the ages of 15 and 49 died from pregnancy-related problems in 2014 alone, with these sub-Saharan Africa accounts for 65% (179,000) of the deaths. Although studies are conducted on modern contraceptives, information is scarce on multinomial regression analysis at the national level data. Therefore, this study aimed to assess modern contraceptive method utilization and determinant factors among women in Ethiopia.

**Methods:**

Data for this study were extracted from the national representative 2019 Ethiopian Mini Demographic and Health Survey. Data was collected using a 2-stage cluster design, in which enumeration areas formed the first stage and households made the second stage. The survey was conducted from March 21, 2019, to June 28, 2019. The analysis was done using multinomial logistic regression using STATA software version 14. The overall categorical variables with a P value of < 0.25 at the binomial analysis were included in the final model of the multinomial logistic regression model in which odds ratios with 95% CIs were estimated to identify the independent variables of women’s modern contraceptive utilization. P values less than 0.05 were used to declare statistical significance. All analysis was done on weighted data.

**Results:**

A total of 8885 (weighted) participants were included in the current study from these,. The current study revealed that the prevalence of modern contraceptive utilization was 28.1% (95%CI: 27.6.7-28.6%). Factors like: women 25 to 34 years (aRRR = 1.5;95% CI:1.2–1.9), 35 to 44 years (aRRR = 2.4; 95% CI: 3.3–5.4), and greater than 45 years (aRRR = 2.9; 95% CI: 2.2–3.7); place of residence (rural; aRRR = 0.89; 95% CI 0.81–0.99), higher educational status (aRRR = 0.035;95%CI:0.61–0.98), grandmultipara (aRRR = 1.73;95%CI:1.6–1.9), and wealth index (poorer aRRR = 0.541;95%CI:0.46–0.631.9) were the factors significantly associated with the outcome variable.

**Conclusions:**

In this, modern contraceptive utilization is low as compared to other countries. It was influenced by age, place of residence, education, the number of children, and wealth index. This suggests that creating awareness of contraceptive utilization is paramount for rural residence women by policymakers and health managers to empower women for family planning services. Moreover, all stakeholders, including governmental and nongovernmental organizations, better to emphasize on modern contraceptive use.

## **Background**

Globally, sexual and reproductive health is a significant public health issue for women of the reproductive age group [1]. Women from low and middle-income nations (LMICs) have an increased chance of dying from pregnancy-related complications [2]. Family planning methods aid in preventing high-risk pregnancies, unsafe abortions, reproductive tract infections, and sexually transmitted infections (STIs) such as HIV/AIDS [3, 4]. For example, family planning method utilization could avert an anticipated 218 million unintended pregnancies, 55 million spontaneous births, 138 million premature births, and 118,000 maternal deaths in 2012 [5]. Therefore, the use of contraceptives is regarded as a secure and cost-effective method for preventing unwanted pregnancy [6].

Despite its importance, nearly 200 million women do not utilize safe and effective family planning methods globally [2, 7, 8]. Globally, Sub-Saharian African (SSA) countries have the highest average fertility rate [9–13]. However, family planning method utilization is the lowest in this region, with particular issues among youthful ladies between the ages of 15 and 24 [14]. For example, approximately 222 million women would like to delay or stop childbearing but are not using any contraception in SSA [5, 15]. Generally, contraceptive prevalence rate among married women has been increased from 14.7 to 29.6% in the last two decades [5, 16]. Family planning success is typically measured by two key family planning indicators: the modern contraceptive prevalence rate (mCPR) and the unmet need for contraception [17] .

Ethiopia is one of the SSA with the highest fertility rates. According to the Ethiopian Demographic Health Survey (EDHS) 2016 report, the total Fertility Rate (TFR) was estimated as 4.6 children per woman [18]. Between 2015 and 2030, the time period of the 2030 Agenda for Sustainable Development, contraceptive use is projected to increase particularly in countries where less than half of reproductive age groups currently use contraception [19] .Specifying that the international community should assess the ‘Proportion of women of reproductive age (ages 15–49 years) who have their need for family planning satisfied with modern methods’. One proposed benchmark related to SDG 3.7.1 is to have at least 75% of the demand for family planning satisfied with modern contraceptives in all countries by 2030 [20]. In line with this vision, Ethiopia has committed increasing the modern contraceptive prevalence rate from 35 to 55% amongst married women by 2020 and reducing the total fertility rate from 4.6 to 3.0 [21]. The variations of modern contraceptive use among sexually active women in Ethiopia are influenced by different variables including socio-economic characteristics, health system factors, source of information, behavioral and attitudinal factors [22]. knowledge [23] parity and mass media [24]. Although many studies were conducted in Ethiopia about contraceptive utilization among women in the reproductive age group, there is no national,organized and comprehensive data which can show all types of family planning options in a multinomial model. This can help stakeholders to look for different strategies for better policy and practice in improving family planning utilization. Therefore, this study aimed to assess the prevalence and factors associated with modern contraceptive use among women in the reproductive group Ethiopia .

## Methods

### Setting area

Ethiopia is the biggest and most crowded nation in East Africa. The capital city is Addis Ababa, found nearly within the center of the nation. According to the Central Statstical Agency (CSA) estimation, the current population of Ethiopia is 122,754,582. At the current time, the country has 9 geological districts and 2 cities organizations. Ethiopia encompasses a three-tiered healthcare conveyance framework. A area healing center, health centers, and their today health posts make up the primary level, which is the essential level. A common healing center makes up the moment level of the level, whereas a strength clinic makes up the third. Both are solely dedicated to giving corrective healthcare administration.

### Study design

This study used data from the Ethiopia Demographic and Health Survey(EDHS) 2019. EDHS 2019 is the fifth and most recent demographic and health data, conducted from Walk 21, 2019, to June 28, 2019. EDHS 2019 gives a point by point examination of Ethiopia’s general populace, child well-being, and maternal issues employing a cross-sectional consideration. For the purpose of the study, the women’s recode files, which contain data on women aged 15–49 years were used. The DHS is a nationally representative survey that is conducted in over 85 low- and middle-income countries and focuses on important maternal, and child health markers such as contraceptive use [25].

### Data source

The DHS program office gave an authorization letter to access the 2019 EDHS, which is the fourth comprehensive survey. EDHS 2019 included a total of 9150 households from 9 regions / of 9150 households, only 8,663 were successfully interviewed. A full protocol explaining the data collection process and sampling method of EDHS 2019 can be reviewed elsewhere. The dataset of this study can be found from the measure of the DHS : https://dhsprogram.com/pubs/pdf/FR363/FR363.pdf.

### Population and sampling procedures

All women in the reproductive age group were the source population in this study. All women aged 15 to 49 years who lived in the selected households, and presented during data collection period were the study population. Each household was considered as the sampling unit. Besides, each woman was taken as the study unit. Each region was stratified into urban and rural clusters, which yielded a total of 21 sampling strata. Multistage cluster sampling was applied in EDHS 2019. In the first stage, a total of 305 Enumeration Areas (EAs) (93 in urban areas and 212 in rural areas) were selected randomly and allocated proportionally to the household size from the sampling strata. Second, a total of 30 households per cluster were selected with an equal probability using systematic random sampling. Finally, a representative samples of 9150 households were selected in 2019 EMDHS, of which 8,794 households were occupied during data collection. Of the occupied households, only 8,663 households were interviewed successfully, yielding a response rate of 99%. A total of 8885 eligible women ,whose age was from 15 to 49 year, were included in the analysis of this study. Simple random sampling strategy was followed to select one woman if there were more than one eligible women in the household. The detailed explanation of the sampling procedure can be found in the methodology of the EDHS 2019 final report [26].

### Data collection tool and procedures

EDHS used structured and pre-tested questionnaire as a tool for data collection. Structered interview schedules were performed by trained interviewers. Frequent interviews were performed using local language. Information about socio-demographic and reproductive characterstics of women were collected using women questionnaire. The 2016 EDHS questionnaire asked all reproductive-age women involved in the survey if they want children in the future to assess their fertility intention. The women responded as they want soon, want later, or want no more. Those women who responded “want no more” were included in this analysis. The outcome variable for this analysis was contraceptive use, which has two categories (yes or no). Similarly, the 2016 EDHS questionnaire asked all reproductive-age women involved in the survey whether they were using contraceptives at the time of survey including the type of contraceptive. If the woman reported she was using contraceptives (modern or traditional), she was considered as a contraceptive user (yes) and otherwise “no”.

### Study variables

The dependent variable was modern contraceptive utilization in this study. It is a categorical variable classified as modern, traditional and no contraceptive. Women who utilized modern contraceptives were labeled as ‘2’,women who used traditional contraceptives were labeled as ‘1’ and women who did not use any method were coded as ‘0’ in this study.

We have included a total of 10 explanatory variables related to contraceptive utilization in this study. They were categorized as socio-demographic variables like residence,wealth index,educational status and reproductive factors, which include the following variables: age, parity,marital status, parity and number of children. household wealth index was calculated using the principal component analysis (PCA) of data on housholds’ ownership of assets like television, radio,mobile computer and car and housing characteristics (access to electicity, source of drinking water, type of floor material,type of toilet facility, number of bed room, and type of cooking fuel. Wealth index scores were adusted scores were adusted for the type of residence (rural or urban). Then, DHS assigned each person in the population his/her household’s score. The study population was ranked according to their social economic scores and divided into 5 groups (20% of the population) known as wealth quintiles.

### Operational definitions

#### Modern contraceptives

Contraceptive types considered as modern methods are male and female sterilization, intrauterine device (IUD), Injectable, oral contraceptive (pills), lactational amenorrhea method (LAM), Standard day’s method (SDM) or a condom).

#### Traditional contraceptives

traditional contraceptive methods were withdrawal and periodic abstain.

#### Knowledge of contraceptives

if women can list at least one type of modern contraceptive method, she can be assumed as knowledgable about contraceptives.

### Data analysis

Data analysis was carried out using STATA version 14; logit based multinomial (polymoutous) logistic regression analysis was computed to distinguish factors associated with contraceptive utilization. Women who took no method were consirdered as a refernce category and specified as ‘0’. On the contrary, the remaining categories were considered as alternate categories. Variance Inflation Factor (VIF) was assessed to check the presence of multicollinearity. VIF value greater than 10 was considered as an indication of multicollinearity. However, no significant multi-colliearity was observed. The parameter of aRRR indicates the likelihood to membership of one category of the independent variable compared to the reference category. The closer the value of AOR to zero, the less effect the explanatory variable has on the dependent variable’s alternative categories as compared to the reference category. Descriptive analyses were conducted to describe the characteristics of the study participants and the results were presented using tables and texts. We used the svy commands to account for the complex survey sampling and used sampling weights to account for unequal probability sampling in different strata. Bivariate logistic regression analysis was used to assess the individual relationship of each explanatory variable with women’s modern contraceptive use, while multivariate analysis was used to assess relationships while controlling for other explanatory variables. The overall categorical variables with a P value of < 0.25 in the binary logistic regression analysis were included in the final model of the multinomial logistic regression model in which odds ratios with 95% CIs were estimated to identify the independent variables of women’s modern contraceptive utilization. P values less than 0.05 were used to declare statistical significance. All analysis was done on weighted data. Bivariate logistic regression analysis was performed to show associations between explanatory variables and outcome variable using the chi-square test.Variables which show associations on bivariate logistic regression analysis were exported to multi-variate multinomial logistic regression model.

We thought it was suitable to predict nominal dependent variables (stunting, wasting, and underweight child status) given one or more independent variables (age in months, sex, residence region, mother’s education, and wealth quintile). The explanatory variables were significantly linked with the outcome variables through multivariable logistic regression models, resulting in an odds ratio with 95% confidence intervals. And the p-value was used to test the statistical significance of the predictors with a lower reference point than 0.05. We have applied the backward stepwise selection to remove variables in the final model. We verified the final model fit using the Hosmer-Lemeshow goodness-of-fit test (9.40, p value of 0.31).

## Results

### Socio-demographic characterstics

According to the report of Table 1, a total of 8885 study participants were included in the analysis of this study. Since this study was conducted on secondary data. All of the study participants were included in the final analysis, making a non-response rate of 100%. Majorities of the study participants lived in three main regions in Ethiopia. Specifically, 1052(11.8%), 1008(11.3%), 948(10.7%) of women lived in Oromia, Southern Nations Nationalities, and Peoples, and Amhara regions, respectively. Nearly three-quarters (66.8%) of the study participants were rural dewllers. Nearly half (49.6%) of the study participants did not attend any formal education. About one-third (32.7%) of the study participants were categorized into richest wealth index (**See** Table 1).

**Table 1:** Background characteristics of the study participants, in 2019 (*n* = 8885)

Background characteristics	Weighted Frequency (n)	Weighted Percent (%)
**Region**		
Tigray Afar Amhara Oromia Somali Benishangul SNNPR Gambela Harari Addis Ababa Dire Dawa	733 641 948 1052 640 747 1008 723 763 818 812	8.2 7.2 10.7 11.8 7.2 8.4 11.3 8.1 8.6 9.2 9.1
**Place of residence**		
Urban Rural	2951 5934	33.2 66.8
**Literacy**		
Cannot read at all Able to read only parts of sentence Able to read whole sentence No card with required language Blind/visually impaired	4406 1067 2904 505 3	49.6 12.0 32.7 5.7 0.0
**Educational Level**		
No education Primary Secondary Higher	3640 3345 1149 751	41.0 37.6 12.9 8.5
**Religion**		
Orthodox Protestant Muslim Catholic Traditional Others	3374 78 1711 3635 60 27	38.0 0.9 19.3 40.9 0.7 0.3
**Wealth index**		
Poorest Poorer Middle Richer Richest	2031 1341 1268 1344 2901	22.9 15.1 14.3 15.1 32.7

### Reproductive characteristics

Of 8885 study participants, more than one-fifth (22.6%) were from the age group of 15 to 19 years. Almost one-third (32.6%) of the study participants were nulli-para. About three-fifths (60.4%) of the study participants were married. Nearly one-fourth (23.2%) of the study participants were never in a union till data collection period. Majorities (95%) of study participants have known modern methods of contraception. However, hardly afew(0.001%) study participants knew traditional (folkloric method) (**See** Table 2).

**Table 2:** Reproductive characteristics of the study participants (N = 8885)

Study variables	Count	Percent
**Age**		
15–19	2006	22.6
20–24	1518	17.1
25–29	1677	18.9
30–34	1105	12.4
35–39	974	11.0
40–44	686	7.7
45–49	526	5.9
**Parity**		
None	2897	32.6%
1 to 2	2404	27.1%
3 to 4	1705	19.2%
5 and above	1879	21.1%
**Marital status**		
Never in union	2618	29.5
Married	5365	60.4
Living with partner	105	1.2
Widowed	210	2.4
Divorced	405	4.6
**Knowledge of contraceptive**		
Knows no method	424	4.8
Knows only folkloric method	4	0.001
Knows only traditional method	13	0.1
Knows only modern methods	8884	95

### Prevalence modern contraceptive utilization

The prevalence of modern contraceptive utilization among women of reproductive age was found to be 28.1% (95%CI: 25.6-28.6%) in this study. Moreover,the traditional contraceptive utilization was only 0.7% among women in the reproductive age group in Ethiopia in 2019.

### Multivariate multinomial logistics regression analysis

Statistical analyses were carried out at three levels in the study. At the univariate level, frequency distribution, percentages and tablels were used to describe sample characteristics and contraceptive utilization. At the bivariate level, unadjusted multinomial logistic regression coefficients were used to examine the association (positive or negative) between specific explanatory or control variables and contraceptive utilization. At the multivariate level, the adjusted multinomial logistic regression was used to further examine the association between the research variables. To be selected for inclusion in the multivariate analysis, a variable must have shown statistical significance with at least one outcome category at the bivariate level (p-value less than 0.25). Multinomial logistic regression analysis is most suitable for the study because the three categories of the outcome variable do not have natural ordering. If the categories are ordered, the ordered logistic regression will also be suitable for the study. However, the shortcoming of the ordered logistic regression is that it will not provide separate regression coefficients for each category of the outcome variable; hence the multinomial logistic regression was preferred.The statistical significance of the *F*-statistics for the goodness of fit suggests that the models fit the data well.

Based on the report of Table 3,a total of 10 variables showed significant association in bi-variate logistic regression analysis. They were further fitted to a multivariate multinomial logistic regression model based on the category of the response variable. Two models were fitted by categories such as traditional method vs. no method, and the modern contraceptive vs. no method, and its confidence interval was used. A P-value of 0.05 or lower indicates the significance of the outcome variable. Women in the age group 25–34 years, 35-44years, and 45 + years increase the relative risk of modern contraceptive use as compared to women aged between 15 and 24 years in the modern contraceptive vs. no method category (aRRR = 1.5,95%CI:1.2,1.9),(aRRR = 95%CI: 3.3–5.4), (aRRR = 1.9,95%CI = 2.2,3.7) respectively. Rural women decrease the relative risk of using both traditional and modern contraceptives when compared with urban in both traditional vs. no method and modern contraceptive vs. no method (aRRR = 0.37,95%CI:0.22–0.62) and (aRRR = 0.032,95%CI: 0.81,0.99) respectively. Holding other variables constant, the chance of educated women increases, relative to the risk of using traditional (aRRR = 0.101, 95% CI: 0.05,0.28) contraceptives and modern contraceptive (aRRR = 0.405,95%CI: 0.19–0.83). Furthermore, keep other variables constant, unmarried women decrease the relative risk of using contraceptives in both categories compared with their counterparts (aRRR = 0.06,95%: 0.046–0.26) and (aRRR = 0.06,95%: 0.046–0.26) respectively.

**Table 3:** Multivariable multinomial regression analysis of contraception methods (N = 8,885)

Study variables	Contraceptive utilization					
	Traditional method vs. No method			Modern contraceptive vs. No method		
	**RRR**	**P-value**	**%CI**	**RRR**	**P-value**	**CI**
Age						
15–24 years	-	-	-	**Ref**	**Ref.**	**Ref.**
25–34 years	-	-	-	1.5	0.001	1.2–1.9
35–44 years	-	-	-	4.2	0.000	3.3–5.4
45^+^ year	-	-	-	2.9	0.001	2.2–3.7
Residence						
urban	**Ref**		**Ref.**		**Ref.**	**Ref.**
rural	0.37	0.000	0.22–0.62	0.89	0.032	0.81–0.99
Educational status						
No education	**Ref**		**Ref.**		**Ref.**	**Ref.**
Elementary school	0.101	0.000	0.046–0.28	0.82	0.06	0.67–1.01
secondary	0.405	0.014	0.19–0.83	0.87	0.191	0.71–1.07
Higher	0.477	0.093	0.20–1.31	0.77	0.035	0.61–0.98
Religion						
Orthodox	**Ref.**		**Ref.**		**Ref.**	**Ref.**
Catholic	0.162	0.002	0.051–0.51	0.043	1.661	1.02–2.71
Protestant	0.360	0.000	0.065–0.47	0.008	2.784	1.3–5.92
Muslim	0.181	0.005	0.055–0.59	0.052	1.632	0.99–2.67
Traditional	0.240	0.014	0.077–0.75	0.689	0.904	0.55–1.48
Sex of the household						
Male	-	-	-		**Ref.**	**Ref.**
Female	-	-	-	0.000	2.5	2.22–2.92
Marital status						
Married	**Ref.**		**Ref**		**Ref**	**Ref**
Single	0.06	0.000	0.046–0.26	0.000	0.061	0.05–0.07
Number of children in the household						
1–5	**Ref**		**Ref.**		**Ref.**	**Ref**
more than 5	0.82	0.39	0.51–1.29	1.73	0.000	1.6–1.9
Wealth index						
poorest	**Ref.**		**Ref.**		**Ref.**	**Ref.**
Poorer	0.298	0.003	0.13–0.67	0.541	0.000	0.46–0.63
middle	0.096	0.000	0.026–0.35	0.737	0.000	0.64–0.85
richer	0.273	0.003	0.118–0.63	0.991	0.901	0.86–1.13
richest	0.440	0.015	0.228–0.85	0.866	0.034	0.75–0.98

**Key: RRR = Relative Risk Ratio, CI = Confidence Interval, Ref = Reference**

Women who had more than five family members increase the relative risk of using traditional methods compared with women who had less than or equal to five children (aRRR = 1.73,95%: 1.6 ,1.9). The relative risk of using traditional was decreased among women in the category of poorer middle and richer(aRRR = 0.298,95%CI:0.13–0.67), (aRRR = 0.273,95%CI:0.118–0.63) and (aRRR = 0.44,95%CI:0.228–0.85) respectively in traditional vs. no method category.

The relative risk of using traditional was decreased among women in the category of poorer middle and richer(aRRR = 0.541,95%CI:0.46–0.63),(aRRR = 0.737,95%CI:0.64–0.85),and (aRRR = 0.866,95%CI: 0.75–0.98), respectively as compared to women in modern contraceptive vs. no method category (**See** Table 3).

## Discussion

In this study, we used nationally representative, population based survey data-of the Ethiopia Demographic and Health Survey. It included nearly 8885 women in the reproductive age group. The prevalence of modern contraceptives was estimated as 28.1% among women in the age group of 15–49 years. The current finding was higher than findings of studies conducted in Liberia (23.87%) [5], Burkina Faso (23.6%) [27],and Hadiya, Ethiopia (23.9%) [28]. Furthermore, it is lower than findings of study findings done in Ethiopia (36.7%) [29], Zambia (43%) [30], and South Africa (54.67%) [31]. The possible rationale for the discrepancies might be due to a difference in awareness of modern contraceptive methods. Besides, it might also be due to the sociocultural differences among different countries. This implies that there are higher fertility and infant mortality rates [5]. The other rationale could be because of the educational status of women and their beliefs about using contraception. On the other hand, the prevalence of traditional contraceptives was only 0.07%. This finding is extermely lower than findings of studies in other countries [32, 33]. The possible reason for this difference might be due to variation in social action and community perception [34]. Furthermore, the variation in knowledge of traditional contraceptive method,side effects, the bad experience with the modern contraceptive methods, spousal communication around family planning, the husband’s opposition to modern methods, availability, accessibility [35].Women found in the age group of 25–34 years had higher odds of using modern contraceptive use compared with younger women aged 15–24 years. This is similar to the study done in African countries [36]. The possible explanation for this variation could be that women aged 15 to 24 years might not have a better understanding of the consequences of engaging in unprotected sex or the benefits of modern contraceptive use as compared with women aged between 25 and 34 years [37, 38]. Contraceptive use increases with young age and decreases with old age, which is consistent with reports from Malawi and Ethiopia [39, 40]. Because, old aged women in their menopausal stage mostly have decreased sexual activity and are less likely to use contraceptives.

This study finding indicates that women residing in rural areas were less likely to use modern contraceptive methods than urban women. This is in line with a study done by Juniper Russo (2014) and Mtuy and Mahande (2015), on Afghanistan [41], Pakistan [42], and two areas of Bangladesh [43], [1]. The reason for the difference may be that rural women have less access to contraceptive services and other maternal healthcare services [44]. Besides, the observed differences might be due to differences in cultural views and beliefs about the use of contraceptives [37]. For example, some women believe that using contraceptives is sinful. As a result, they don’t use them. Additionally, urban women are more likely to use any kind of contraception and have better access as a whole [45]. The other possible reasons for high use of urban dwellings include the better socioeconomic status of women and cultural disparity. Furthermore, limited access to and availability of family planning services in rural areas might further limit the use of modern contraceptives. Single women were less likely to use modern contraceptives.

In this study, women who had more children were more likely to use modern contraceptives compared to their counterparts. This is in agreement with a study done in East African countries [46], which claimed that women who have more children are more likely to use a modern contraceptive method. The rationale could be for the achievement of a desired family size, which is a reliable indicator of willingness to use modern contraceptive methods. This factor strongly connects with the desire to have no more children and is indicative of the achievement of desired family size. In this study, Muslim, Catholic, and protestant religious followers were less likely to use modern contraceptives than the orthodox Christian religion. This finding is in line withfindings of studies done in Kersa, Eastern Ethiopia [47] and Ghana [48]. The acceptance of family planning methods by religion is crucial, yet participants’ views of their faith’s position on this matter varied [49]. As a result, it implies that individuals may prioritise using contraceptives over holding to their religious views as long as they are aware of the advantages, such as avoiding unintended births and sexually transmitted diseases [4]. This may be because many Muslims feel that family planning is forbidden in the Holy Book, as noted in another report [50]. Educated women were more likely to use contraceptives than their counterparts.This finding is consistent with findings of studies in Ethiopia [51–53]. The possible reason might be due to that many countries now support women’s education both to foster economic growth and also to promote reasonable family sizes, and improve child health and women’s sexual reproductive health [54]. This is not astonishing because education supports women to be well informed on the benefits of contraceptives and empowers them to have the independence to make decisions on their fertility and in the exercise of their reproductive rights [37].The findings of the current study indicate that women with a low level of wealth index have better chance of using contraceptive than their counterparts. This finding is consistent with finding of study done in Ethiopia [55]. On the contrary,this finding is in contrary with the prevous findings of study in Ethiopia [56, 57]. The possible variation could be due to differences in their fertility needs. In Ethiopia, modern contraceptives are available free of charge, the contribution of household wealth to contraceptive use will not be explained by the ability to pay for the service. Rather, it reflects the general socioeconomic position of the household [58].

### Strengths and limitations of the study

The results of this study can be generalized due to its large sample size, national study and use of standard questionnaires.In addition, the method of sampling and weighing suggests the strength of this study. However, this study has many limitations. For example, it was a cross-sectional study. Therefore, this study could not conclusively determine the temporal relationship between explanatory and outcome variables. In addition, several cultural and health institutional factors were not investigated in this study.

## Conclusions

In this, modern contraceptive utilization is low as compared to others. It was influenced by age, place of residence, education, the number of children, and wealth index. This suggests that creating awareness towards contraceptive utilization is important specially for rural residence women by policymakers and health managers to empower women for family planning service. Moreover, all stakeholders, including governmental and nongovernmental organizations, better to emphasize on modern contraceptive use.

### Implication of the study

This study coun have many public health implications. First, this study highlights the benefit of developing and implementing policy to improve knowledge of the impact of contraceptives. strategies should be further designed to enhance modern family planning for those women with low socio-economic status.

## Data Availability

Permission to access database was ofcially obtained. The database was available at a ofcial website of DHS which is at https://dhsprogram.com.

## Abbreviations

MOR: Multinomial Odds Ratio
CI: Confidence Interval
DHS: Demographic Health Survey
PCA: Principal Component Analysis
LMICS: Low and Middle Income Countries
HIV: human immune virus
AIDS: Acquired Immune Defficinecy Syndrome
SDG: Sustanaible Development Goal
